# A dataset of distribution and diversity of blood-sucking mites in China

**DOI:** 10.1038/s41597-021-00994-9

**Published:** 2021-08-05

**Authors:** Fan-Fei Meng, Qiang Xu, Jin-Jin Chen, Yang Ji, Wen-Hui Zhang, Zheng-Wei Fan, Guo-Ping Zhao, Bao-Gui Jiang, Tao-Xing Shi, Li-Qun Fang, Wei Liu

**Affiliations:** State Key Laboratory of Pathogen and Biosecurity, Beijing Institute of Microbiology and Epidemiology, Beijing, 100071 People’s Republic of China

**Keywords:** Entomology, Geography, Epidemiology

## Abstract

Mite-borne diseases, such as scrub typhus and hemorrhagic fever with renal syndrome, present an increasing global public health concern. Most of the mite-borne diseases are caused by the blood-sucking mites. To present a comprehensive understanding of the distributions and diversity of blood-sucking mites in China, we derived information from peer-reviewed journal articles, thesis publications and books related to mites in both Chinese and English between 1978 and 2020. Geographic information of blood-sucking mites’ occurrence and mite species were extracted and georeferenced at the county level. Standard operating procedures were applied to remove duplicates and ensure accuracy of the data. This dataset contains 6,443 records of mite species occurrences at the county level in China. This geographical dataset provides an overview of the species diversity and wide distributions of blood-sucking mites, and can potentially be used in distribution prediction of mite species and risk assessment of mite-borne diseases in China.

## Abstract (summary)

**Table:** 

Measurement(s)	Distribution
Technology Type(s)	digital curation • Geographic Information System
Factor Type(s)	genus • species • province • city • county • GPS
Sample Characteristic - Organism	mite • Acari
Sample Characteristic - Location	China

Machine-accessible metadata file describing the reported data: 10.6084/m9.figshare.14459055

## Background & Summary

Vector-borne infections (VBI) are defined as infectious diseases transmitted by the bite or mechanical transfer of arthropod vectors. They constitute a significant proportion of the global infectious disease burden. Ticks and mosquitoes are recognized as the most important vectors in the transmission of bacterial and viral pathogens to humans and animals worldwide^1^. High priority of VBIs mainly include malaria, dengue, chikungunya virus infection, Zika virus disease, lymphatic filariasis, Lyme disease, tick-borne spotted fever and tick-borne encephalitis, etc., which are primarily transmitted by ticks or mosquitoes, while mite-borne infections were often considered as neglected diseases. According to the recent studies, many infectious diseases transmitted by the bite or mechanical transfer of mite vectors^2–4^, including scrub typhus^5^, hemorrhagic fever with renal syndrome (HFRS)^6^ and plague^7^, are of global importance. Moreover, mites can parasitize vertebrates, including livestock, birds, rodents, and other wild animals^8–10^, with intrinsic vector competence to transmit various pathogens of medical and veterinary importance^11,12^. Therefore, mites have caused a major threat to human and animal health.

Currently, more than 55,000 species of mites are known to exist in the world^13^, with complex living environment in both outdoor and indoor, which differed in accordance with their hosting habitats. Mites usually have a wide geographical distribution with low host specificity. Mammals, birds, reptiles, amphibians and even some other arthropods are reported to be the hosts of mites^14^. The larvae of mites are often found on the body surface of small mammals, especially rodents (rats, mice and voles) which are the most common hosts of mites^15^. For instance, more than 30 species of small mammals and birds have been reported to be the hosts of *Leptotrombidium delicense*^16^. The feeding habits of mites are complicated. Some of the domestic mite species found in indoor environments and in warm or tropical regions, such as house dust mite, are well known for causing allergic disorders and not to suck blood. Most of the parasitic mites with medical importance are related to human and animal and are blood-sucking^17–19^. Gamasid mites, chigger mites and oribatid mites are among the most commonly seen mite species that bite and transmit pathogens^20^.

The Chinese climatic and environmental conditions provide suitable conditions for many mite species, among which a large number of mite species are blood-sucking, having negative influences on human and animal health. Against a true baseline estimate of the geographic distributions of mite species, we can then prepare for the new disease threats associated with mite-bite. However, most of the recent studies have been carried out to report the identification of novel mite species^21^, the novel endemic regions^22^, novel mite-borne diseases^6^, or even the genomics and developmental transcriptomics analyses of certain mite species^23^. For those reports on the geographic distributions of mite species, the publication language was usually in Chinese. The geographic scope was rather limited and recorded in a patchy way^24^, or with low spatial resolutions and representing an aggregate of geographical points and time points^25^. The available dataset is far from adequate to attain a complete understanding of the distributions of mites in China. Moreover, many historical collection of mite-related records were documented in Chinese, and the largest two datasets were (China Economic Entomology, 《中国经济昆虫志》^26^; Atlas of Epidemiology of Natural Focus Diseases in China, 《中国自然疫源性疾病流行病学图集》^27^) compiled by Institute of Zoology, Chinese Academy of Sciences and our institute (Beijing Institute of Microbiology and Epidemiology). A study on breaking language barriers for a more complete picture of mite landscapes in China would be useful for future disease risk analysis and modelling experiments. To better identify the geographic distributions of the major mite species in China, we reviewed the prevalence, geographical distributions, and zoonotic potential of blood-sucking mites in China. Both English and Chinese records were reviewed, which were collectively composed into the dataset that comprised 6,443 records of geo-referenced mite occurrence without repetition at county level reported from 1978 to 2020, involving 551 mite species in 759 locations across China. The most frequently reported mite species are *Haemolaelaps glasgowi* and *Laelaps echidninus*.

## Methods

### Data Collection

The aim of the data assembly was to build a comprehensive database of reports on the distributions of mites in the mainland of China, with the study subject focusing on the blood-sucking mites. A systematic literature review on the distributions of blood-sucking mites in China was performed according to the Preferred Reporting Items for Systematic reviews and Meta-Analyses (PRISMA) statement guidelines^28^. The literature search was conducted in both Chinese and English literature databases, mainly through China National Knowledge Infrastructure (CNKI) (http://www.cnki.net/), Wan Fang Database (http://www.wanfangdata.com.cn/), VIP Database (http://www.cqvip.com/), PubMed Database (https://pubmed.ncbi.nlm.nih.gov/), related domestic zoological works and research data on the distribution of mites in our laboratory over the years. After the first-round literature search, we made double check to find the publications that were overlapped in three Chinese databases (CNKI, Wan Fang database, and VIP database). There might also be duplicated references in PubMed with Chinese core journal database, which were checked by the method provided in Giovanny Herrera *et al*.^29^, and with the English literature included only. Although the literature search was made during the period from 1950 to 2020, the earliest one can be traced back to 1978 and the latest was reported on October 15^th^, 2020. The terms (“螨” or “螨虫”) were used in Chinese journal databases, and (‘mite’ and ‘China’) were used in PubMed search. No language restrictions were placed on these searches.

**Fig. 1:**
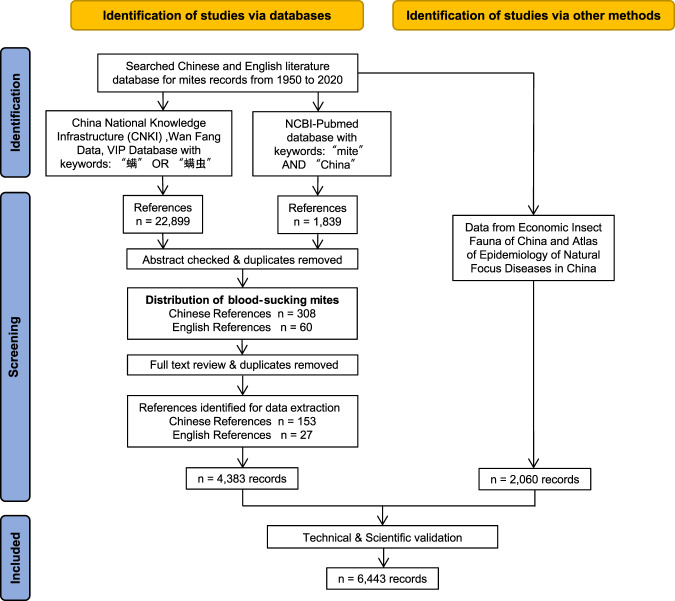
Schematic diagram of the literature search process and results.

**Fig. 2:**
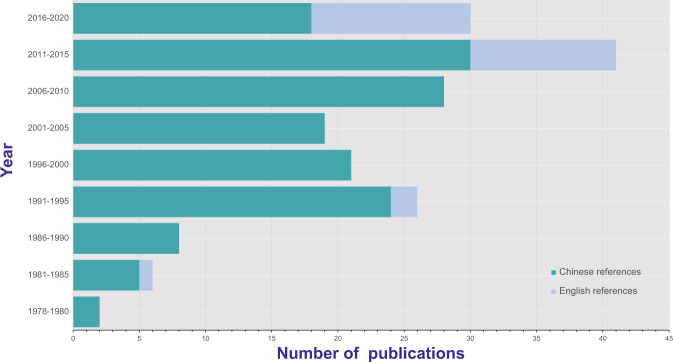
Numbers of publications that recorded mite obtained from Chinese and English references. An abrupt increase of publications with mite records was seen after 1990.

To attain a valid information extraction, we followed the three-step guideline for data screening. For the first step screening, totally 24,738 publications, including 22,899 Chinese and 1,839 English abstracts were retrieved for initial screening using the key words searching. For the second-step screening, the abstracts of all returned references were reviewed to exclude those reporting only clinical case or laboratory data and without mentioning any specific mite species. For the third step screening, we reviewed the full text of all the 308 Chinese references and 60 English references in detail, from which a total of 153 Chinese references and 27 English references were determined to be eligible for data extraction (Fig. 1). The earliest Chinese and English publications were published in 1978 and 1990, respectively, and an abrupt increase of references that reported mite records was seen after 1990 (Fig. 2).

**Fig. 3:**
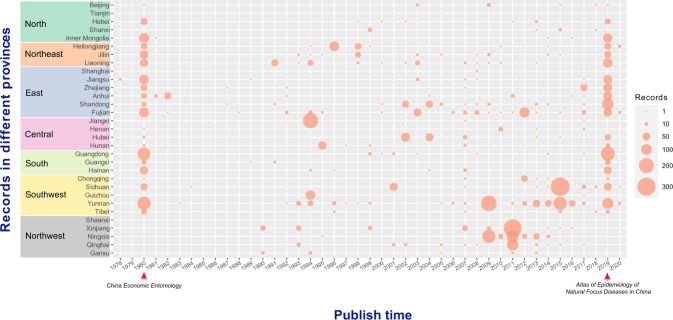
The number of reported records for mites in different provinces between 1978 and 2020. The bubble diagram was used with the size of bubble indicating the number of records. The abrupt increase of publications in 1980 and 2019 was due to the supplementation of the data collected from the two books in Chinese (Economic Entomology and Atlas of Epidemiology of Natural Focus Diseases in China).

The key data we extracted from the literature included: (i) name of the mite genera, (ii) name of the mite species, (iii) the reported geographic location information (province, city and county), (iv) time of publication, (v) source of literature. After the initial data entrance, a twice re-check by two researchers was performed to avoid errors and duplicates. Each record represents the distribution of one mite species in one certain district. Among them, 4,101 records were extracted from Chinese journals and 282 records from English journals. In addition, 2,060 records were extracted from China Economic Entomology and Atlas of Epidemiology of Natural Focus Diseases in China, two books that have involved a large number of field surveys since 1980 in 31 provinces (autonomous regions and municipalities) across the country, which have supplemented the distribution of mites in the mainland of China. In total, 6,443 records were compiled at the county level, and differences of the number of records between provinces and over years were shown in Fig. 3.

### Geo-positioning

For each record of mite, we extracted the location information at the county level from the selected references. Since the multiple surveys in the same county may be reported in different publications for different research purposes, we summarized all the records reported in the references and deleted the duplicate records of the same species at the same location. For the references that reported no longitude and latitude of the study sites, we used the ArcGIS Desktop 10.7.0.10450 software to extract the geographical coordinates of the center points of the corresponding administrative areas from the digital map, China administrative boundary maps (2015), which were obtained from the Resource and Environment Data Cloud Platform, Chinese Academy of Sciences (http://www.resdc.cn/data.aspx?DATAID=202/). In order to accurately demonstrate the spatial precision of each record, we included the ‘location_level’ field in the dataset to ensure that each location was pinned into the correct administrative regions. The standardized data were uploaded to the figshare website for potential data set users to download^30^. We use RStudio Version 1.2.5001 and ArcGIS Desktop 10.7.0.10450 software to statistically analyze and visualize the obtained geographic data.

## Data Records

The dataset of distributions and diversity of blood-sucking mites in China is available on figshare^30^. Each row of the dataset represents a distinct record, which records one specific mite species in a specific location by a scientific literature at a specific time. The columns contained in the dataset are shown as follows:

1. **Genus:** Identifying the genus of mites.
2. **Species:** Identifying the species of mites.
3. **Province:** Provincial level information of mites reported location, including names of 34 provinces, autonomous regions, municipalities or special administrative regions of China.
4. **City:** City level information of mites reported location, including names of 333 prefectural-level cities or autonomous prefectures.
5. **County:** County level information of mites reported location, including names of 382 counties.
6. **Province_code:** Adopt 2018 China’s administrative division province name code.
7. **City_code:** Adopt 2018 China’s administrative division city name code.
8. **County_code:** Adopt 2018 China’s administrative division county name code.
9. **GPS_xx:** Longitude of reported county coordinates.
10. **GPS_yy:** Latitude of reported county coordinates.
11. **Location_level:** The geographic scale of location (1 = provincial level, 2 = city level, 3 = county level).
12. **Duplicate name:** If species name has duplicate names (0 = no, 1 = yes).
13. **Data source:** Details about whether location data were extracted from literature data or research site survey data (1 = Chinese literature data, 2 = English literature data, 3 = data from China Economic Entomology and Atlas of Epidemiology of Natural Focus Diseases in China).
14. **Publish_time:** The year of publication.
15. **References:** The full name of references identified for data extraction.

## Technical Validation

**Fig. 4:**
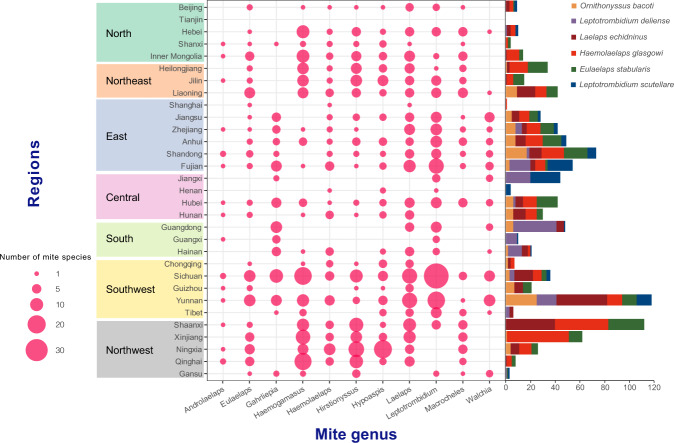
The number of blood-sucking mite species of eleven main mite genera reported in different provinces with the size of red bubble indicating the number of mite species. The bar chart indicates the distribution of 6 major mite species that carry pathogens.

This dataset contains 6,443 records that were extracted from 153 Chinese references and 27 English references, as well as from China Economic Entomology and Atlas of Epidemiology of Natural Focus Diseases in Chinese. All records were extracted and entered under the same standard by the same person at the initial stage. The data were subsequently double checked by another member, with the emphasis on screening the duplicate data and correcting the ambiguous data, and supplementing the missing data. Data were strictly checked to ensure that accuracy and extraction criteria were met, by an approach similar to that used in Herrera *et al*.^29^. In the process of confirming the geographic location information, an independent third-party was designated to re-check the geographic location information of the data. The verification process refers to the same standard in the data entry process to re-check the accuracy of the data, by a similar approach to that used in Battle *et al*.^31^. In order to unify the geographic location information that was extracted from references with no uniform standard to the county level, ArcGIS software was used to determine the coordinates of the central points of the counties, which were marked on Baidu Map (https://map.baidu.com/) to ensure that each coordinate point corresponds to an accurate administrative region in China. The resulting locations of mite occurrence as depicted in Fig. 4 agree well with the previous findings and maps.

Due to the wide time span of the literature search (1978‒2020), the classification and naming of some mite species have changed. After a thorough re-evaluation, the standard synonymisations were followed: *Hypoaspis* (*Laelaps*) *pavloskii* as synonym of *Androlaelaps pavlovskii*^32^; *Laelaps jettmari* as synonym of *Laelaps huaihoensis*^33^; *Hypoaspis freemani*, *Haemolaelaps magaventralis* and *Haemolaelaps haemorrhagicus* as synonyms of *Haemolaelaps casalis*^34^; *Haemolaelaps microti*, *Haemolaelaps morhrae* and *Haemolaelaps scalopi* as synonyms of *Haemolaelaps glasgowi*^34^; *Hyperlaelaps microti* as synonym of *Hyperlaelaps aravalis*^34^; *Hypoaspis murinus* and *Hypoaspis smithii* as synonym of *Hypoaspis* (*Geolaelaps*) *lubrica*^34^; *Cosmolaelaps gurabensis* as synonym of *Hypoaspis* (*Cosmolaelaps*) *miles*^34^; *Hypoaspis sinicus* as synonym of *Hypoaspis* (*Geolaelaps*) *praesternalis*^34^; *Haemolaelaps angustiscutis* as synonym of *Haemolaelaps fragilis*^35^; *Haemogamasus macrodentilis* as synonym of *Haemogamasus daliensis*^36,37^; *Proctolaelaps pygmaeus* as synonym of *Hypoaspis ovatus*^38,39^; *Proctolaelaps fiseri* as synonym of *Proctolaelaps yinchuanensis*^40^; *Pachylaelaps siculus* as synonym of *Pachylaelaps xinghaiensis*^41,42^.

## Usage Notes

Blood-sucking mites are important vectors of infectious diseases of global concern. Being aware of the distribution of blood-sucking mites is of great significance to prevent and manage relevant diseases. To the best of our knowledge, this dataset represents the first and the most comprehensive database for the blood-sucking mite species, together with their distribution at multiple scales in China. Compared to previous studies, this database contains a total of 6,443 pieces of data, recording the distributions of 551 species of mites from 759 counties that spanned from 1978 to 2020. To make the dataset easily filter or aggregate for investigation purposes and methodologies, each record of the mites was paired with relevant geo-positioning and timeline variables.

The dataset is helpful for understanding the spatial distributions, ecological niches, and geographic co-occurrence patterns of mite species, and can also be used to identify the spatial profile of mite-borne pathogens, with expectation that future health impacts of the mite-borne infections could be projected, and better surveillance and control of mite-borne diseases can be guided in China. The diagrams created in the paper might give a straightforward clue as to which kind of pathogen could be carried by the mites at specific locations. According to the current data review, *Leptotrombidium delicense* (*L. delicense*), *Leptotrombidium scutellare* (*L. scutellare*), *Ornithonyssus bacoti* (*O. bacoti*), *Laelaps echidninus* (*La. echidninus*), *Haemolaelaps glasgowi* (*H. glasgowi*) and *Eulaelaps stabularis* (*E. stabularis*) are the most frequently reported blood-sucking mites with competence to transmit various pathogens, which were widely distributed in China (Fig. 4). *L. delicense* and *L. scutellare* are the main vectors of *Orientia tsutsugamushi* (*O. tsutsugamushi*)^43,44^, which have been found in more than 15 provinces of China. Both trombiculid and gamasid mites, including *O. bacoti*, *La. echidninus*, *H. glasgowi* and *E. stabularis* are naturally infected with Hantavirus, which might effectively transmit the virus^45^. *L. scutellare* mites, the vector of *O. tsutsugamushi*, had been tested positive for *Richettsia* by PCR or RT-PCR amplification, including *Richettsia felis*, *Richettsia australis* and an unnamed *Rickettsi*a sp. TwKM02, according a report from Shandong Province in China^46^.

Additionally, this dataset can be extrapolated and used to guide estimation of the global clinical burden of mite-borne diseases, which is largely unknown. Although some mite species are often limited to specific geographical regions, there might have geographic expansion that increase their potential of re-location elsewhere, probably due to international travel from endemic regions to non-endemic regions by people, animals and cargo shipment or climate change. The contemporary distributions and mapping of the mite species on a fine spatial scale would also benefit the prediction of future trends and possibility of transmission in unaffected regions by applying model construction. This dataset is crucial for developing innovative strategies to control mites and mite-borne diseases.

## Data Availability

No custom code was made during the collection and validation of this dataset.
